# Construction and validation of machine learning models for sepsis prediction in patients with acute pancreatitis

**DOI:** 10.1186/s12893-023-02151-y

**Published:** 2023-09-01

**Authors:** Fei Liu, Jie Yao, Chunyan Liu, Songtao Shou

**Affiliations:** 1 Department of Emergency Medicine, Tianjin Medical University General Hospital, 154 Anshan Road, Heping District, Tianjin, 300052 P.R. China; 2 Department of Anesthesiology, The First Affiliated Hospital of Hebei North University, Zhangjiakou, Hebei 075000 P.R. China; 3 Department of Intensive Care Unit, The First Affiliated Hospital of Hebei North University, Zhangjiakou, Hebei 075000 P.R. China

**Keywords:** Machine learning models, Logistic regression model, Scoring systems, sepsis, Acute pancreatitis

## Abstract

**Background:**

This study aimed to construct predictive models for the risk of sepsis in patients with Acute pancreatitis (AP) using machine learning methods and compared optimal one with the logistic regression (LR) model and scoring systems.

**Methods:**

In this retrospective cohort study, data were collected from the Medical Information Mart for Intensive Care III (MIMIC III) database between 2001 and 2012 and the MIMIC IV database between 2008 and 2019. Patients were randomly divided into training and test sets (8:2). The least absolute shrinkage and selection operator (LASSO) regression plus 5-fold cross-validation were used to screen and confirm the predictive factors. Based on the selected predictive factors, 6 machine learning models were constructed, including support vector machine (SVM), K-nearest neighbour (KNN), multi-layer perceptron (MLP), LR, gradient boosting decision tree (GBDT) and adaptive enhancement algorithm (AdaBoost). The models and scoring systems were evaluated and compared using sensitivity, specificity, positive predictive value (PPV), negative predictive value (NPV), accuracy, and the area under the curve (AUC).

**Results:**

A total of 1, 672 patients were eligible for participation. In the training set, 261 AP patients (19.51%) were diagnosed with sepsis. The predictive factors for the risk of sepsis in AP patients included age, insurance, vasopressors, mechanical ventilation, Glasgow Coma Scale (GCS), heart rate, respiratory rate, temperature, SpO2, platelet, red blood cell distribution width (RDW), International Normalized Ratio (INR), and blood urea nitrogen (BUN). The AUC of the GBDT model for sepsis prediction in the AP patients in the testing set was 0.985. The GBDT model showed better performance in sepsis prediction than the LR, systemic inflammatory response syndrome (SIRS) score, bedside index for severity in acute pancreatitis (BISAP) score, sequential organ failure assessment (SOFA) score, quick-SOFA (qSOFA), and simplified acute physiology score II (SAPS II).

**Conclusion:**

The present findings suggest that compared to the classical LR model and SOFA, qSOFA, SAPS II, SIRS, and BISAP scores, the machine learning model-GBDT model had a better performance in predicting sepsis in the AP patients, which is a useful tool for early identification of high-risk patients and timely clinical interventions.

**Supplementary Information:**

The online version contains supplementary material available at 10.1186/s12893-023-02151-y.

## Background

Acute pancreatitis (AP), an inflammatory disease of the pancreas, is the leading cause of hospital admissions for gastrointestinal diseases worldwide [1, 2]. The worldwide incidence rate of AP is 33.74 per 100,000 person-years, with a gradual increase in incidence [3, 4]. Approximately 10–20% of patients with AP have complicated systemic inflammatory response syndrome (SIRS) and multiple organ dysfunction syndrome, which can lead to the development of severe AP with a mortality rate of 10–15% [5]. Sepsis is a life-threatening SIRS caused by the host’s dysregulated response to infection, which ultimately leads to septic shock and multiple organ failure and is the main cause of health loss all over the world [6]. Up to 40–70% of patients with AP will develop an infection related to pancreatitis in the late stages, or sepsis in severe cases [7, 8]. The progression of AP to sepsis is associated with higher mortality rates and a poor prognosis [9]. Therefore, early identification of AP patients who are likely to develop sepsis is of great significance for reducing mortality and disease burden.

Several scoring systems have been identified to predict the severity and prognosis of AP and sepsis, including the SIRS score, bedside index for severity in acute pancreatitis (BISAP) score, sequential organ failure assessment (SOFA) score, quick-SOFA (qSOFA), simplified acute physiology score II (SAPS II) [10–12]. However, poor performances of scoring systems in predicting sepsis have been observed [13]. The predictive performance of the logistic regression (LR) model based on conventional clinical indicators in predicting sepsis among patients with AP was also moderate, with the area under the receiver (AUC) of the operating characteristic curve (ROC) value being 0.73 [9]. Advanced machine learning algorithms can model nonlinear relationships, analyze complex high-order interactions, and robustly handle multicollinearity among the predictor variables [14]. Machine learning has been widely used in the diagnosis/risk prediction of sepsis, and the prognosis of sepsis. A database study conducted a machine learning approach to predict 30-day mortality for patients with sepsis, the AUC of the model was 0.857 [15]. A study conducted in the Chinese population used a machine learning model for accurate prediction of sepsis in intensive care unit (ICU) patients, the established machine learning-based model showed good predictive ability with AUC being 0.91 [16]. In addition, the machine learning model also showed excellent predictive value for severe AP and concurrent acute kidney injury (AKI) risk in AP [17, 18]. However, to the best of our knowledge, no study has reported the application of machine learning in predicting the risk of sepsis in patients with AP. The early detection and prediction of patients who may develop sepsis are essential to improve the adverse consequences of AP.

Herein, this study aimed to (1) construct predictive models for the risk of sepsis in patients with AP using machine learning methods and validate the predictive performances; (2) select the optimal machine learning model and compare it with the LR model and scoring systems. This study may help to identify the risk of sepsis in patients with AP at an early stage and assist in the clinical treatment of AP and the prevention of sepsis.

## Methods

### Data design and study population

This study was a retrospective cohort study. Data were collected from Medical Information Mart for Intensive Care III (MIMIC III) database (https://mimic.mit.edu/docs/iii/) between 2001 and 2012 and the MIMIC IV database (https://mimic.mit.edu/docs/iv/) between 2008 and 2019. MIMIC-III includes data from more than 58,000 admissions to Beth Israel Deaconess Medical Center in Boston from 2001 to 2012, including 38,645 adults and 7,875 neonates [19], and MIMIC-IV includes 524,740 admissions for 382,278 patients at this center from 2008 to 2019 [20, 21]. The included criteria were (1) aged ≥ 18 years; (2) diagnosed with AP upon intensive care unit (ICU) admission. Excluded criteria were (1) patients with a length of ICU stay less than 24 h; (2) diagnosed as sepsis upon ICU admission. he requirement of ethical approval for this was waived by the Institutional Review Board of Tianjin Medical University General Hospital, because the data was accessed from MIMIC III database and MIMIC IV database (publicly available database). The need for written informed consent was waived by the Institutional Review Board of Tianjin Medical University General Hospital due to retrospective nature of the study. All methods were performed in accordance with the relevant guidelines and regulations.

### Data extraction

Data collected from the database including (1) baseline characteristics: age (years), gender (male), Race (Black, White, and other), insurance (government, private, and unknown), marital status (divorced, married, separated, single, widowed, and unknown), interventions (vasopressors, mechanical ventilation), and effusion; (2) vital signs: heart rate (bpm), respiratory rate (breaths/minute), temperature (°C), SpO_2_ (%), systolic blood pressure (SBP, mmHg), diastolic blood pressure (DBP, mmHg); (3) scoring systems: SOFA score, qSOFA score, SAPS II score, BISAP, SIRS, Glasgow Coma Scale (GCS), charlson comorbidity index (CCI), International Normalized Ratio (INR); (4) laboratory values: white blood cell (WBC, K/uL) count, platelet count (K/uL), hemoglobin (g/dL), red blood cell distribution width (RDW, %), hematocrit (%), bilirubin (mg/dL), blood creatinine (mg/dL), prothrombin time (PT, sec), partial thromboplastin time (PTT, sec), blood urea nitrogen (BUN, mg/dL), glucose (mg/dL), calcium (mg/dL), sodium (mEq/L), chloride (mEq/L), and total bicarbonate (mEq/L). All the data were extracted from the data generated within the first 24 h after the patient entered the ICU.

### Variable definition

Patients diagnosed with AP were determined by using the International Classification of Diseases (ICD) (ninth edition, code 577.0 or 10th version, code K 85.0) codes. Sepsis was diagnosed according to the sepsis-3 criteria [22]; in brief, patients with documented or suspected infection and an acute change in total SOFA score of ≥ 2 points were considered to have sepsis. Infection was identified from the ICD code.

SOFA score calculated the dysfunction of six organ systems and the severity of the dysfunction, including the respiratory, coagulation, liver, cardiovascular, kidney, and nervous systems with a score of 0–4 for each item and a total score of 0–24 [23]. qSOFA score: calculated by the presence of changes in mental status, respiratory rate > 22 breaths per minute, and preoperative systolic blood pressure < 100 mmHg [22]. The SAPS II score (0-163) consists of 17 variables composed of 12 physiological variables, age, type of admission, and three different underlying disease variables [24]. Components of the BISAP scoring system included BUN > 25 mg/dl, impaired mental status, SIRI, age > 60 years, and pleural effusion [25]. SIRS was defined as two or more out of the following four: temperature > 38.0 °C or < 36.0 °C, heart rate > 90 beats/minute, respiratory rate > 20 breaths/minute, leukocytosis > 12,000/dL, or leucopenia < 4,000/dL [26].

### Outcome and follow-up

The outcome of the study was the risk of sepsis. Follow-up was conducted during hospitalization in the ICU and the end point of follow-up was sepsis or discharge from the ICU. The mean follow-up time was 3.64 (1.93–9.70) days.

### Construction and performances assessment of the machine learning models

The patients were randomly divided into two groups, of which 80% were used as the training set and the remaining 20% as the testing set. Based on the predictive factors selected, 6 machine learning models were constructed including support vector machine (SVM), K-nearest neighbor (KNN), multi-layer perceptron (MLP), LR, gradient boosting decision tree (GBDT), and adaptive enhancement algorithm (AdaBoost). The models were evaluated and compared by sensitivity, specificity, positive prediction value (PPV), negative prediction value (NPV), accuracy, and the AUC of the ROC.

### Sample size calculation for predictive models

Our sample size calculation aimed to ensure a precise estimation of model parameters while minimizing the potential of overfitting. In order to achieve the goal of an average absolute prediction error (MAPE) of 0.05, as suggested by Riley et al. [27], 478 samples would be sufficient for a maximum of 13 predictors, a statistically determined risk prediction model.

### Statistical analyses

Variables with more than 20% missing values were excluded from further analysis. Random forest imputation was used to deal with missing data below 20%. Random forest imputation is a nonparametric algorithm that accommodates nonlinearities and interactions and does not require the specification of a specific parametric model [28]. Supplementary Table 1 shows the variables with missing values below 20%. Sensitivity analysis was performed to compare the data before and after imputation (Supplementary Table 2). Means ± standard deviations (SD) was used to describe the distribution of normally distributed measurement data, and T-test was used to compare the differences between the two groups. Medians and quartiles were used to represent measurement data that conformed to a normal distribution, and rank-sum tests were used for comparisons between groups. Count data were expressed as the number of cases and composition ratio (%), and the chi-square test was used for comparison between groups.

The least absolute shrinkage and selection operator (LASSO) (“LassoCV” method in Sklearn) regression plus 5-fold cross-validation were used to screen and confirm the predictive factors and selected the best alpha = 0.0075 when one standard error of the minimum mean squared error (MSE) was used as a screening criterion. In order to select the optimal model from the 6 machine learning models, Delong’s test was used. Comparing the performance of the optimal machine learning model with LR, scoring systems (SOFA, qSOFA, SIRS, SAPS II, and BIASP). Clinical benefit was assessed using Decision Curve Analysis (DCA). A *P* < 0.05 was considered statistically significant. Python 3.9.0 (Python Software Foundation) and R (version 4.2.2) were used for all analyses.

## Results

### Basic characteristics of the study population

**Fig. 1:**
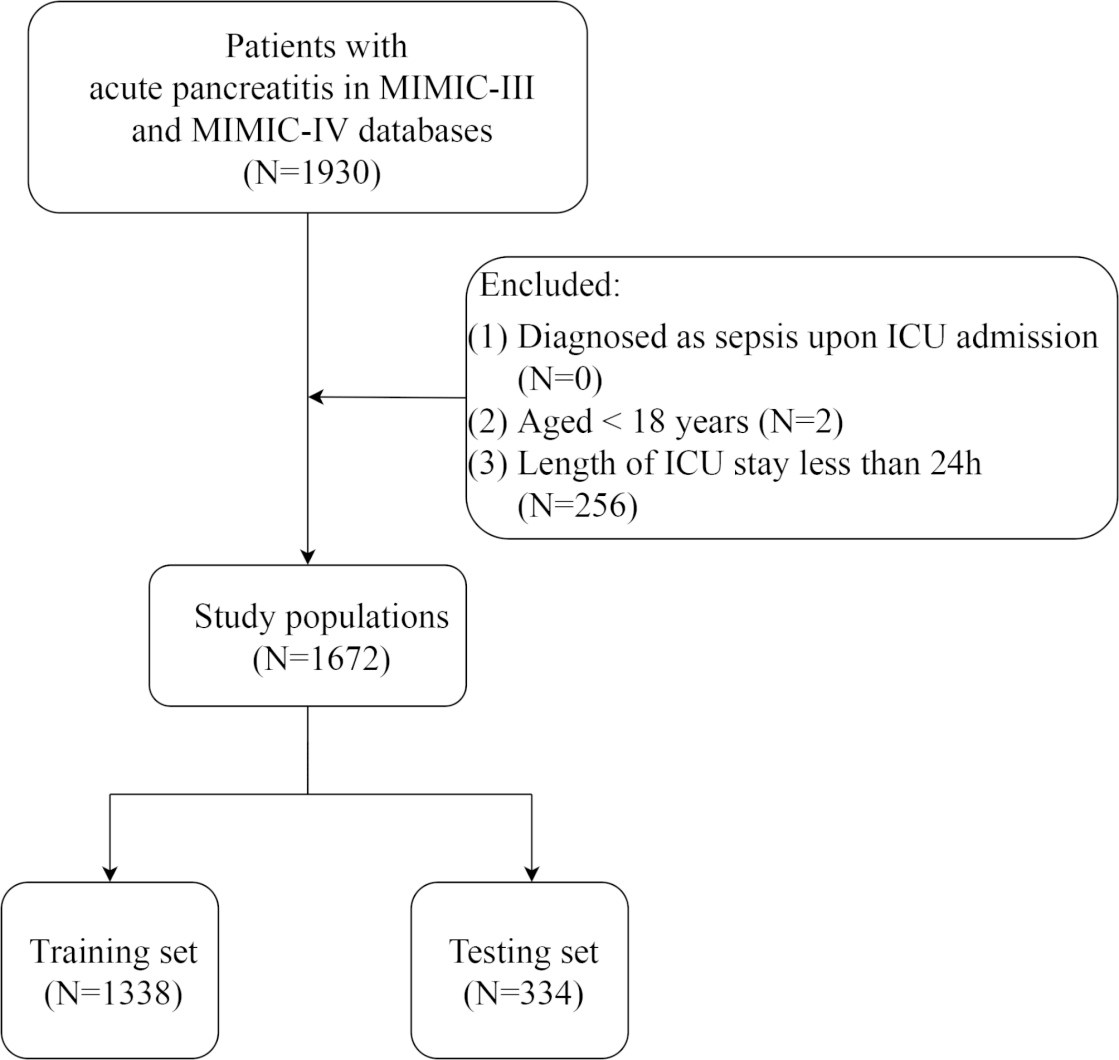
The flow chart of the participants selection

A total of 1,930 participants diagnosed with AP were screened from MIMIC III and MIMIC IV databases; of these 1,930 patients, 256 were excluded due to the length of ICU stay less than 24 h, and 2 were excluded due to the age < 18 years. Finally, 1, 672 patients were eligible for participation, with 1,338 patients in the training set and 334 patients in the testing set. The flow chart of the participants’ selection is depicted in Fig. 1. In the training set, 261 AP patients (19.51%) were diagnosed with sepsis. In the training set, the mean age of the AP patients with sepsis was 58.43 (16.50) years, 58.2% of the AP patients with sepsis were male, 42.9% of the AP patients with sepsis were married, 67.8% of the AP patients with sepsis were on vasopressors, and 95.8% of the AP patients with sepsis were on mechanical ventilation. The mean GCS score of AP patients with sepsis was 9.73 (4.48), the mean heart rate of AP patients with sepsis was 102.91 (23.15) bpm, and the mean respiratory rate of AP patients with sepsis was 22.57 (6.86) breaths/minute. There were significant differences between AP patients with and without sepsis in insurance, marital status, vasopressors, mechanical ventilation, GCS, heart rate, SBP, respiratory rate, SpO_2_, WBC, RDW, blood creatinine, BUN, bicarbonate, SOFA, qSOFA, SAPS II, SIRS, and BISAP (each *P* < 0.05). All baseline characteristics of the study population are summarized in Table 1.

**Table 1:** Basic characteristics of study population

Variables	The training set (n = 1338)				The testing set (n = 334)		
	Sepsis				Sepsis		
	No (n = 1077)	Yes (n = 261)	*P*		No (n = 268)	Yes (n = 66)	*P*
Age, years, (mean (SD))	58.73 (17.48)	58.43 (16.50)	0.802		59.30 (17.01)	57.10 (16.36)	0.342
Gender (male), n (%)	603 (56.0)	152 (58.2)	0.557		152 (56.7)	36 (54.5)	0.857
Ethnicity, n (%)			0.071				0.21
Black	108 (10.0)	30 (11.5)			26 (9.7)	2 (3.0)	
Other	257 (23.9)	78 (29.9)			72 (26.9)	20 (30.3)	
White	712 (66.1)	153 (58.6)			170 (63.4)	44 (66.7)	
Insurance, n (%)			< 0.001				< 0.001
Government	603 (56.0)	131 (50.2)			144 (53.7)	31 (47.0)	
Private	212 (19.7)	21 (8.0)			61 (22.8)	5 (7.6)	
Unknown	262 (24.3)	109 (41.8)			63 (23.5)	30 (45.5)	
Marital status, n (%)			0.021				0.137
Divorced	86 (8.0)	9 (3.4)			15 (5.6)	8 (12.1)	
Married	452 (42.0)	112 (42.9)			113 (42.2)	26 (39.4)	
Separated	2 (0.2)	2 (0.8)			2 (0.7)	0 (0.0)	
Single	333 (30.9)	85 (32.6)			99 (36.9)	18 (27.3)	
Unknown	87 (8.1)	31 (11.9)			21 (7.8)	10 (15.2)	
Widowed	117 (10.9)	22 (8.4)			18 (6.7)	4 (6.1)	
Vasopressors (yes), n (%)	288 (26.7)	177 (67.8)	< 0.001		73 (27.2)	48 (72.7)	< 0.001
Mechanical ventilation (yes), n (%)	695 (64.5)	250 (95.8)	< 0.001		165 (61.6)	62 (93.9)	< 0.001
GCS, (mean (SD)	13.74 (2.28)	9.73 (4.48)	< 0.001		13.54 (2.48)	8.61 (4.18)	< 0.001
CCI, M (Q_1_, Q_3_)	3.00 (1.00, 5.00)	3.00 (1.00, 5.00)	0.489		3.00 (1.00, 5.00)	2.50 (1.00, 5.00)	0.074
Heart rate, bpm, mean (SD)	98.44 (20.56)	102.91 (23.15)	0.002		97.54 (21.12)	107.39 (25.32)	0.001
SBP, mmHg, mean (SD)	128.35 (25.65)	122.89 (27.50)	0.002		129.56 (24.97)	123.82 (28.57)	0.105
DBP, mmHg, mean (SD)	70.97 (18.71)	69.38 (18.99)	0.221		71.21 (18.68)	70.71 (21.83)	0.852
Respiratory rate, breaths/minute, mean (SD)	20.82 (6.35)	22.57 (6.86)	< 0.001		20.77 (6.36)	21.83 (7.87)	0.249
Temperature, °C, mean (SD)	36.85 (1.02)	36.87 (1.10)	0.824		36.88 (1.06)	37.22 (1.08)	0.018
SpO_2_, % (mean (SD)	96.33 (4.22)	95.41 (5.38)	0.003		96.44 (3.39)	96.32 (3.44)	0.800
WBC, K/uL, (mean (SD)	13.69 (8.29)	15.58 (9.16)	0.001		14.44 (14.24)	15.33 (9.20)	0.631
Platelet, K/uL, (mean (SD)	216.55 (128.30)	217.84 (140.82)	0.887		213.74 (124.85)	202.53 (131.16)	0.518
Hemoglobin, g/dL, mean (SD)	11.14 (2.23)	11.28 (2.50)	0.391		11.26 (2.15)	10.80 (2.53)	0.135
RDW, %, mean (SD)	15.05 (1.95)	15.50 (2.59)	0.002		15.04 (1.93)	15.23 (2.27)	0.502
Hematocrit, %, mean (SD)	33.26 (6.49)	34.03 (7.56)	0.095		33.70 (6.44)	32.70 (7.49)	0.278
Bilirubin, mg/dL, mean (SD)	2.59 (3.80)	2.91 (5.01)	0.265		2.57 (4.83)	3.38 (6.17)	0.248
Blood creatinine, mg/dL, M (Q_1_, Q_3_)	1.00 (0.70, 1.70)	1.30 (0.80, 2.30)	< 0.001		1.10 (0.70, 1.80)	1.55 (0.90, 2.65)	0.008
INR, mean (SD)	1.61 (0.98)	1.65 (1.16)	0.546		1.60 (1.31)	1.58 (0.78)	0.907
PT, sec, mean (SD)	16.89 (8.17)	17.76 (10.78)	0.15		16.74 (10.18)	17.19 (7.47)	0.733
PTT, sec, mean (SD)	35.89 (18.54)	37.36 (19.23)	0.255		32.78 (9.68)	35.88 (10.94)	0.024
BUN, mg/dL, mean (SD)	27.75 (23.91)	32.85 (25.48)	0.002		30.07 (28.00)	39.58 (31.88)	0.017
Glucose, mg/dL, mean (SD)	154.65 (117.42)	167.09 (109.81)	0.12		144.87 (101.00)	141.29 (52.40)	0.781
Calcium, mg/dL, mean (SD)	7.11 (2.44)	6.81 (2.51)	0.073		7.07 (2.49)	6.44 (2.79)	0.074
Sodium, mEq/L, mean (SD)	138.25 (5.35)	138.67 (6.00)	0.265		138.55 (5.89)	138.53 (7.10)	0.983
Chloride, mEq/L, mean (SD)	104.88 (6.99)	105.05 (7.43)	0.728		105.31 (7.45)	105.91 (7.40)	0.561
Bicarbonate, mEq/L, mean (SD)	21.45 (5.53)	20.54 (5.80)	0.018		21.48 (5.29)	20.24 (6.01)	0.098
Effusion = 1 (%)	118 (11.0)	31 (11.9)	0.753		42 (15.7)	8 (12.1)	0.595
SOFA, M (Q_1_, Q_3_)	5.00 (3.00, 7.00)	9.00 (6.00, 13.00)	< 0.001		5.00 (2.00, 7.00)	11.00 (7.00, 14.75)	< 0.001
qSOFA, (Q_1_, Q_3_)	2.00 (1.00, 2.00)	2.00 (2.00, 3.00)	< 0.001		2.00 (1.00, 2.00)	3.00 (2.00, 3.00)	< 0.001
SAPS II, M (Q_1_, Q_3_)	33.00 (24.00, 44.00)	44.00 (32.00, 56.00)	< 0.001		33.00 (23.00, 44.00)	49.00 (33.75, 60.00)	< 0.001
SIRS, M (Q_1_, Q_3_)	3.00 (2.00, 4.00)	3.00 (3.00, 4.00)	< 0.001		3.00 (2.00, 4.00)	3.00 (3.00, 4.00)	0.033
BISAP, M (Q_1_, Q_3_)	2.00 (2.00, 3.00)	3.00 (2.00, 3.00)	< 0.001		2.00 (2.00, 3.00)	3.00 (2.00, 4.00)	< 0.001

Notes: GCS: Glasgow coma scale; CCI: Charlson comorbidity index; WBC: White blood cell count; Platelet: Platelet count; RDW: Red blood cell distribution width; INR: International normalized ratio; PT: Prothrombin time; PTT: Partial thromboplastin time; BUN: Blood urea nitrogen; SOFA: Sequential organ failure assessment score; qSOFA: Quick SOFA; SAPSII: Simplified acute physiology score II; SIRS: Systemic inflammatory response syndrome; BISAP: Bedside index of severity in acute pancreatitis; M: Median; SD: Standard deviations; Q1: 25% Quantile; Q3: 75% Quantile

### Predictive factors selection for the risk of sepsis in AP patients

**Fig. 2:**
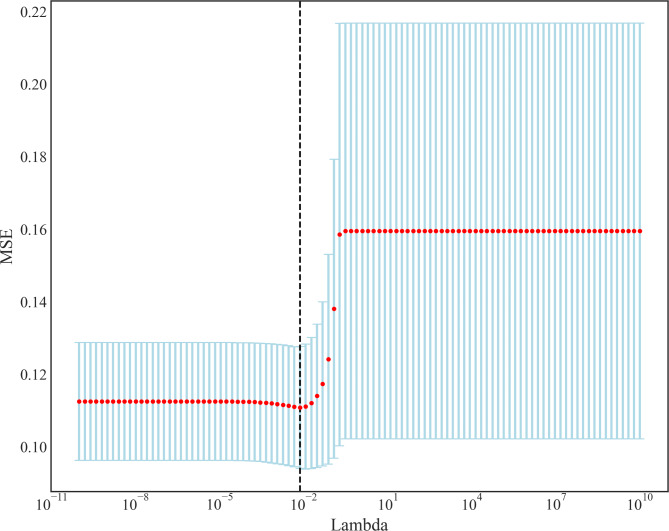
The loss curves for the MSE loss with different Lambda

**Fig. 3:**
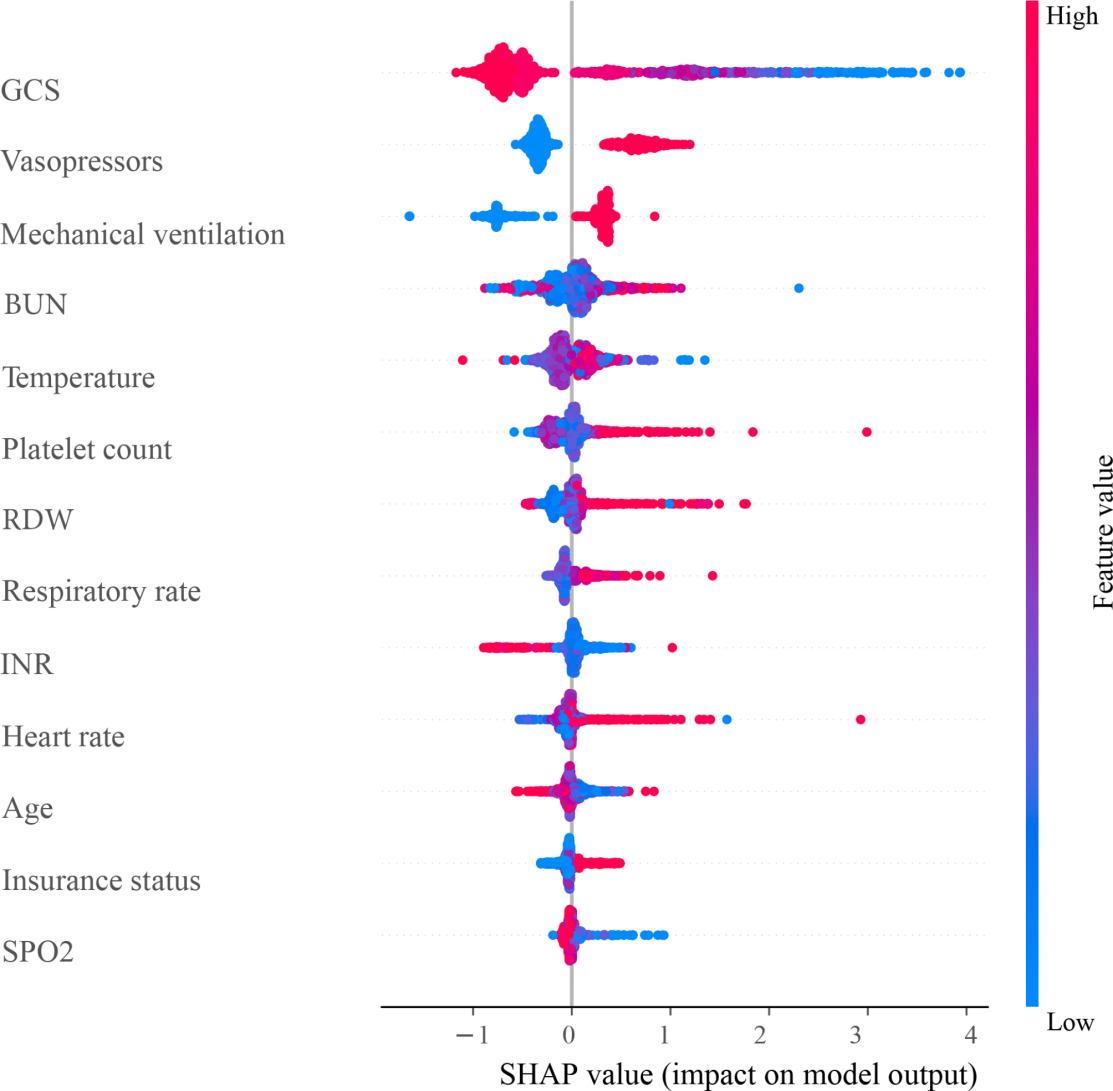
The SHAP plot of the relationship between the value of features and their impact on the model prediction

After LASSO regression selection with 5-fold cross-validation via minimum criteria, 13 variables remain as the predictive factors for the risk of sepsis in AP patients: age, insurance, vasopressors, mechanical ventilation, GCS, heart rate, respiratory rate, temperature, SpO_2_, platelet, RDW, INR, and BUN. Figure 2 shows the loss curves for the MSE loss with different Lambda. The SHAP plot (Fig. 3) shows the relationship between the value of features and their impact on the model prediction. Each row represents the SHAP value distributions of a feature, and the x-axis refers to the SHAP value, where the value of SHAP > 0 shows that the prediction favors the positive class, and a value < 0 indicates that the prediction tends to be the negative class. The color of sample points in Fig. 3 indicates the corresponding feature value: redder points mean higher feature importance values, while bluer points indicate lower feature values. The features are sorted according to the sum of SHAP values incorporating all the samples in the dataset.

### Construction and performance validations of machine learning models

Based on the predictive factors, 6 machine learning models were constructed. The AUC value in the training set of the GBDT model was 0.994 [95% confidence interval (CI): 0.988 to 1.000], higher than the AUC value of the LR model (0.890, 95% CI: 0.860 to 0.920), AdaBoost model (0.918, 95% CI: 0.894 to 0.941), SVM model (0.912, 95% CI: 0.888 to 0.936), KNN model (0.908, 95% CI: 0.883 to 0.933), and MLP model (0.948, 95% CI: 0.929 to 0.967). In the testing set, GBDT had the highest AUC value (0.985, 95% CI: 0.966 to 1.000), thereby, GBDT was selected as the final predictive model. The ACU of the GBDT model (0.985, 95% CI: 0.966 to 1.000) was higher than the LR model (0.896, 95% CI: 0.841 to 0.951), achieving statistical significance (*P* < 0.001). Comparisons of predictive performances among machine learning models are shown in Table 2.

**Table 2:** Construction and performance validations of machine learning models

Methods	AUC (95% CI)	Delong test	Accuracy (95%CI)	Sensitivity (95%CI)	Specificity (95%CI)	PPV (95%CI)	NPV (95%CI)
In training set							
GBDT	0.994 (0.988-1.000)	Ref	0.994 (0.988-1.000)	0.996 (0.989-1.000)	0.992 (0.982-1.000)	0.992 (0.982-1.000)	0.996 (0.989-1.000)
LR	0.890 (0.860–0.920)	< 0.001	0.834 (0.802–0.866)	0.810 (0.762–0.857)	0.858 (0.815-0.900)	0.852 (0.808–0.896)	0.817 (0.771–0.863)
AdaBoost	0.918 (0.894–0.941)	< 0.001	0.918 (0.894–0.941)	0.962 (0.939–0.985)	0.873 (0.833–0.914)	0.885 (0.848–0.922)	0.958 (0.932–0.983)
SVM	0.912 (0.888–0.936)	< 0.001	0.912 (0.888–0.936)	0.924 (0.892–0.956)	0.900 (0.864–0.936)	0.903 (0.868–0.939)	0.921 (0.888–0.954)
KNN	0.908 (0.883–0.933)	< 0.001	0.908 (0.883–0.933)	0.916 (0.883–0.950)	0.900 (0.864–0.936)	0.903 (0.867–0.938)	0.914 (0.880–0.948)
MLP	0.948 (0.929–0.967)	< 0.001	0.948 (0.929–0.967)	0.958 (0.934–0.982)	0.938(0.909–0.968)	0.940 (0.912–0.969)	0.957 (0.932–0.982)
In testing test							
GBDT	0.985 (0.966-1.000)	Ref	0.969 (0.940–0.999)	1.000 (1.000–1.000)	0.940 (0.884–0.997)	0.941 (0.885–0.997)	1.000 (1.000–1.000)
LR	0.896 (0.841–0.951)	< 0.001	0.763 (0.691–0.836)	0.828 (0.736–0.921)	0.701 (0.592–0.811)	0.726 (0.624–0.828)	0.810 (0.709–0.911)
AdaBoost	0.940 (0.900–0.980)	0.099	0.939 (0.898–0.980)	0.984 (0.954-1.000)	0.896 (0.822–0.969)	0.900 (0.830–0.970)	0.984 (0.952-1.000)
SVM	0.924 (0.879–0.970)	0.031	0.924 (0.878–0.969)	0.953 (0.901-1.000)	0.896 (0.822–0.969)	0.897 (0.825–0.969)	0.952 (0.900-1.000)
KNN	0.924 (0.878–0.970)	0.030	0.924 (0.878–0.969)	0.938 (0.878–0.997)	0.910 (0.842–0.979)	0.909 (0.840–0.978)	0.938 (0.880–0.997)
MLP	0.916 (0.868–0.964)	0.017	0.916 (0.869–0.964)	0.922 (0.856–0.988)	0.910 (0.842–0.979)	0.908 (0.837–0.978)	0.924 (0.860–0.988)

Notes: SVM: Support vector machine; KNN: K-nearest neighbor; MLP: multi-layer perceptron; LR: logistic regression; GBDT: gradient boosting decision tree; AdaBoost: adaptive enhancement algorithm; PPV: Positive predictive values; NPV: Negative predictive values; AUC: Area under curve; CI: confidence interval; Ref: Reference

### Comparisons of the predictive performances of the GBDT model with LR model, SOFA, qSOFA, SAPS II, SIRS, and BISAP scores

**Fig. 4:**
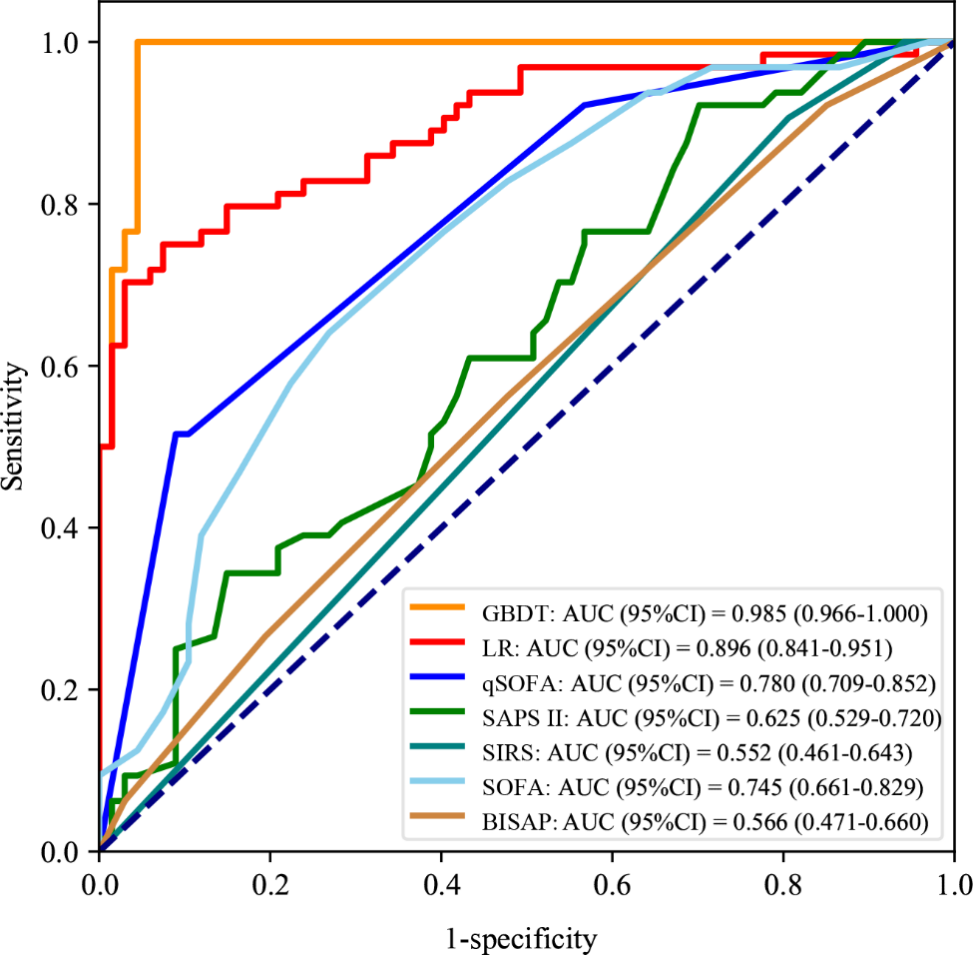
The ROC curve comparison between GBDT and LR models and scoring systems

**Fig. 5:**
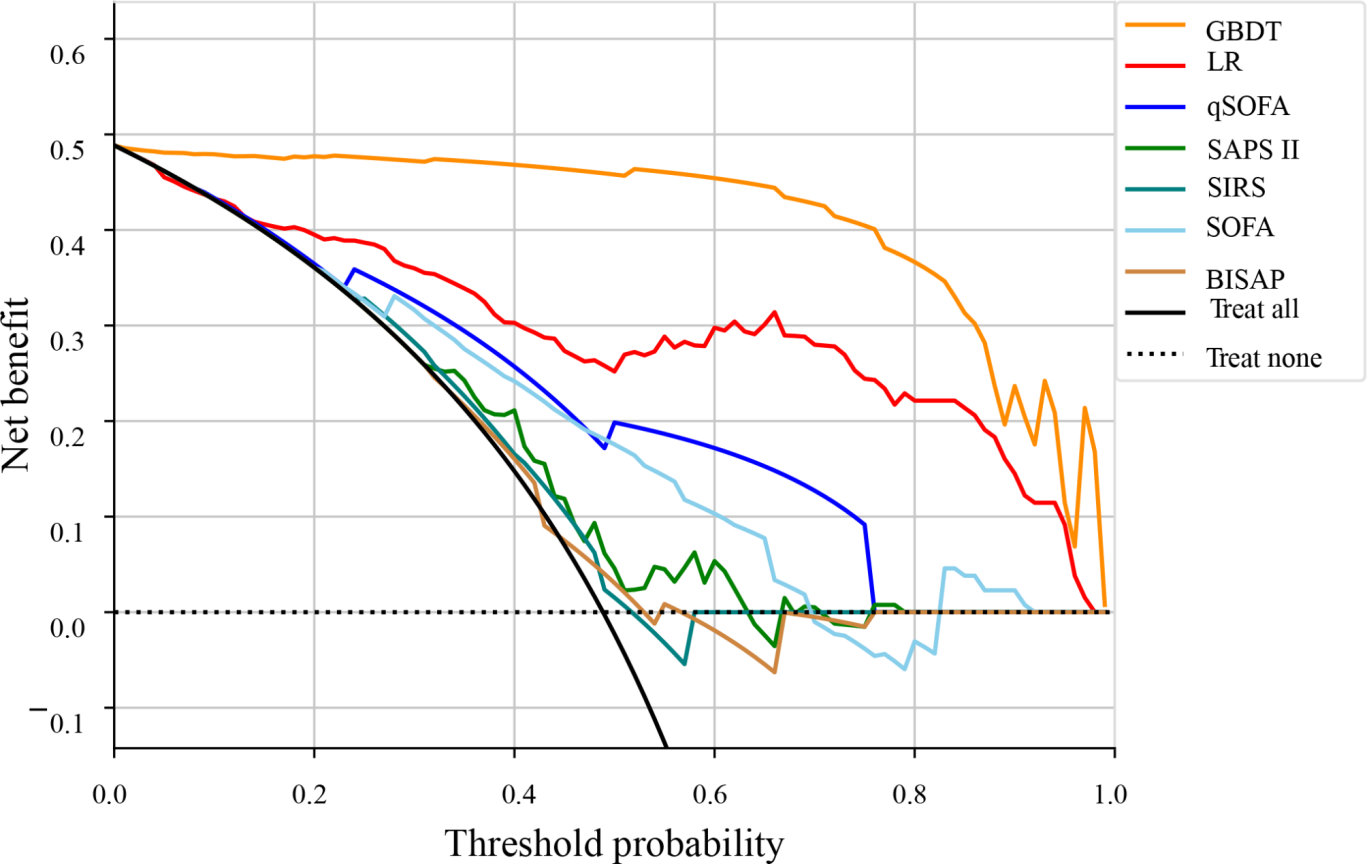
The net benefit of GBDT model, LR model, and scoring systems at different threshold probabilities for predicting sepsis in AP patients

In the testing set, the GBDT model achieved the best performance with an AUC of (0.985, 95% CI: 0.966 to 1.000) compared with qSOFA score (AUC: 0.780, 95% CI: 0.709 to 0.852, *P* < 0.001), SAPS II score (AUC: 0.625, 95% CI: 0.529 to 0.720, *P* < 0.001), SIRS (AUC: 0.552, 95% CI: 0.461 to 0.64, *P* < 0.001), SOFA score (AUC: 0.745, 95% CI: 0.660 to 0.829, *P* < 0.001), and BISAP score (0.566, 95% CI: 0.472 to 0.660, *P* < 0.001). Comparisons of the predictive performances of the GBDT model with SOFA, qSOFA, SAPS II, SIRS, and BISAP scores. Comparisons of the predictive performances of the GBDT model with SOFA, qSOFA, SAPS II, SIRS, and BISAP scores are shown in Table 3. Figure 4 shows the ROC curve comparison between GBDT and LR models and scoring systems. The net benefit for predicting sepsis in AP patients using the GBDT model was greater than the LR model and scoring systems at different threshold probabilities (Fig. 5).

**Table 3:** Comparisons of the predictive performances of the GBDT model with LR model, SOFA, qSOFA, SAPS II, SIRS, and BISAP scores

Methods	AUC (95%CI)	Delong test	Accuracy (95%CI)	Sensitivity (95%CI)	Specificity (95%CI)	PPV (95%CI)	NPV (95%CI)
In the training set							
GBDT model	0.994(0.988-1.000)	Ref	0.994(0.988-1.000)	0.996(0.989-1.000)	0.992(0.982-1.000)	0.992(0.982-1.000)	0.996(0.989-1.000)
qSOFA	0.742(0.703–0.780)	< 0.001	0.673(0.633–0.713)	0.479(0.419–0.539)	0.869(0.828–0.910)	0.787(0.724–0.851)	0.623(0.573–0.672)
SAPSII	0.652(0.605–0.698)	< 0.001	0.587(0.545–0.629)	0.574(0.514–0.634)	0.600(0.540–0.660)	0.592(0.532–0.652)	0.582(0.523–0.641)
SIRS	0.592(0.547–0.637)	< 0.001	0.562(0.520–0.605)	0.452(0.392–0.513)	0.673(0.616–0.730)	0.583(0.516–0.651)	0.549(0.494–0.603)
SOFA	0.761(0.720–0.802)	< 0.001	0.713(0.674–0.752)	0.662(0.604–0.719)	0.765(0.714–0.817)	0.740(0.684–0.796)	0.691(0.638–0.744)
BISAP	0.649(0.605–0.694)	< 0.001	0.614(0.572–0.655)	0.654(0.597–0.711)	0.573(0.513–0.633)	0.608(0.551–0.665)	0.621(0.559–0.682)
In the testing set							
GBDT	0.985(0.966-1.000)	Ref	0.969(0.940–0.999)	1.000(1.000–1.000)	0.940(0.884–0.997)	0.941(0.885–0.997)	1.000(1.000–1.000)
qSOFA	0.780(0.709–0.852)	< 0.001	0.710(0.632–0.788)	0.516(0.393–0.638)	0.896(0.822–0.969)	0.825(0.707–0.943)	0.659(0.562–0.757)
SAPSII	0.625(0.529–0.720)	< 0.001	0.557(0.472–0.642)	0.500(0.378–0.623)	0.612(0.495–0.729)	0.552(0.424–0.680)	0.562(0.448–0.675)
SIRS	0.552(0.461–0.643)	< 0.001	0.527(0.441–0.612)	0.469(0.346–0.591)	0.582(0.464-0.700)	0.517(0.389–0.646)	0.534(0.420–0.649)
SOFA	0.745(0.660–0.829)	< 0.001	0.687(0.608–0.766)	0.641(0.523–0.758)	0.731(0.625–0.837)	0.695(0.577–0.812)	0.681(0.573–0.788)
BISAP	0.566(0.472–0.660)	< 0.001	0.542(0.457–0.627)	0.562(0.441–0.684)	0.522(0.403–0.642)	0.529(0.411–0.648)	0.556(0.433–0.678)

Notes: GBDT: gradient boosting decision tree; SOFA: Sequential organ failure assessment score; qSOFA: Quick SOFA; SAPSII: Simplified acute physiology score II; SIRS: Systemic inflammatory response syndrome; BISAP: Bedside index of severity in acute pancreatitis; PPV: Positive predictive values; NPV: Negative predictive values; AUC: Area under curve; CI: confidence interval; Ref: Reference

## Discussion

In this retrospective study, we developed and validated machine learning-based models for predicting sepsis in AP patients. In the training set, 261 AP patients (19.51%) were diagnosed with sepsis. The results of this study showed that the GBDT model had an excellent performance in the prediction of sepsis in patients with AP, with the AUC in the testing set at 0.985. Furthermore, the GBDT model achieved better predictive performance for sepsis prediction in AP patients compared with the LR model, and scoring systems.

Advanced machine learning methods are good at dealing with high-order interactions and fitting complex nonlinear relationships, and can be used to integrate large amounts of data from electronic health records (EHRs). The application of machine learning to data-driven analysis shows promise for improving predictive performance in healthcare [29–31]. A large retrospective study developed and validated a machine learning tool within 48 h after admission for predicting which patients with AP [32]. A retrospective study enrolling patients with AP from multiple centers explored a machine learning model for early identification of severe AP (SAP) among patients hospitalized for AP, and the model showed evident clinical practicability [17]. The study by Qiu et al. developed and validated three machine-learning models for predicting multiple organ failure in moderately severe and severe AP [33]. A systematic review included 47 machine learning predictive models for AP, with 10 studies reporting severity prediction, 10 studies complication prediction, 3 studies mortality prediction, 2 studies recurrence prediction, and 2 studies surgery timing prediction [34]. The study by İnce et al. evaluated the success of artificial intelligence for early prediction of severe course, survival, and ICU requirements in patients with AP [35]. A meta-analysis suggested that the machine learning approach had a better performance than the existing sepsis scoring systems in predicting sepsis [36]. A systematic review and meta-analysis showed that individual machine learning models can accurately predict sepsis onset ahead of time [37]. A machine learning model for prediction of sepsis in ICU patients showed good predictive ability in Chinese sepsis patients [16]. However, there have been limited studies that constructed predictive models for the risk of sepsis in patients with AP using machine learning methods. This study used machine learning methods to construct predictive models for the risk of sepsis in patients with AP and validated the predictive performance.

The results of this study showed that the GBDT model had an excellent performance in predicting sepsis in AP patients. The GBDT model has been applied to diagnose and predict the outcomes of several diseases. A study that developed and assessed machine learning models for predicting recurrence risk after endovascular treatment in patients with intracranial aneurysms found that the GBDT model showed an optimal prediction performance for predicting recurrence risk in patients with intracranial aneurysms after endovascular treatment in 6 months [38]. Lee et al. established machine learning models for predicting the risk of end-stage renal disease among chronic kidney disease patients who survive sepsis, and the GBDT algorithm yielded an accuracy as high as 0.879, as measured using the AUC [39]. Furthermore, we compared the performance of models, the traditional LR model, and scoring systems to predict sepsis in AP patients in the early stage. The result showed that the GBDT model achieved the best performance in predicting sepsis in terms of the predictive performance. Similarly, a previous study suggested that compared to the classical LR model, machine learning models using features that can be easily obtained at admission had a better performance in predicting AKI in AP patients [40]. A retrospective temporal validation study suggested an interpretable machine learning model performed significantly better than LR and outperformed conventional severity scores in predicting in-hospital mortality among sepsis patients and varying subgroups [31]. The high AUC of the GBDT model, compared to traditional models and scoring systems suggested that machine learning models can be used frequently as an adjunct to clinical decision making and provider’s intuition regarding patient prognosis and ideal next steps in care. Early and effective identification of high-risk patients with sepsis in AP patients can prevent further deterioration of the patient’s condition. This study helps clinicians to develop individualized treatment plans for patients, reducing the disease burden on patients and facilitating the rational allocation of medical resources.

GBDT is an ensemble algorithm widely used for regression and classification tasks. The GBDT algorithm creates multiple weak learners or individual trees by bootstrapping training samples and integrates their outputs to make predictions. The GBDT algorithm is less sensitive to hyperparameters, less prone to overfitting, and easy to implement. For the practical applicability of the GBDT model in a clinical setting, an example of how SHAP can be used locally to explain individual prediction was provided (Supplementary Fig. 1). The GBDT model is a promising approach for sepsis prediction in AP patients, but further research is still needed to evaluate its generalizability to other tasks and its computational efficiency.

This study suggested that the basic characteristics of patients (age, temperature, and insurance) and vital signs (heart rate, respiratory rate, and SpO2 were associated with the risk of sepsis in AP. A study by Hong et al. indicated that age may be useful for predicting the development of persistent organ failure in patients with AP [41]. According to the study by Miller et al., an ED-SAS score that incorporates factors including SpO2 and age provides a rapid method for predicting prognosis in AP [42]. The temperature has been reported as a predictor factor for sepsis in AP patients [9]. heart rate has been observed to be associated with severe AP [43]. The interventions also can predict the risk of sepsis in AP. Early vasopressor use was significantly associated with increased in-hospital mortality among critically ill AP patients [44]. We found that the inflammatory markers including RDW and platelets can predict the risk of sepsis in AP patients. As a part of routine blood tests, RDW is a quantitative measurement of the size variability of peripheral blood red blood cells (RBCs), which reflects the heterogeneity of RBCs. Because the changes in the shape and size of circulating red blood cells are often related to the occurrence and development of hematological diseases, RDW is used for the morphological classification of anemia and differential diagnosis of microcytic anemia [45]. RDW is positively associated with AP severity and is likely a useful predictive parameter of AP severity [46]. Platelets are small pieces of cytoplasm shed by mature megakaryocytes, which participate in the hemostasis function of the body. When the stress effect secondary to acute and critical diseases occurs in the body, the number of platelets will change, and the degree of platelet change will affect sepsis [47]. A study by Feng et al. found that a low platelet count increases the risk of sepsis in patients with AP [9]. Simple, routine, and widespread individual laboratory parameter, BUN has been proposed as marker of disease severity [48]. In this study, BUN could be used to predict the risk of sepsis in AP patients. A study by Hong et al. demonstrated that BUN could predict severe AP [49]. Farrell et al. found that persistent elevation of BUN is associated with the development of severe AP [50]. Previous studies have also suggested that BUN is strongly associated with sepsis [51, 52]. GCS was originally used as an assessment tool for patients with head injuries to assess the coma of patients, which has become an important part of the system to determine the severity of an injury [53]. In this study, GCS could predict the risk of sepsis in patients with AP. A retrospective analysis also demonstrated that GCS was among the predictive factor of sepsis among patients with AP [9].

Our study has several strengths. To the best of our knowledge, we first report the application of machine learning models to predict the risk of sepsis in AP patients using the MIMIC database. The optimal model was screened using a variety of machine learning methods and showed significantly better predictive value than LR and scoring systems, providing a basis for the accurate prediction of sepsis risk in AP patients. The sample size in this study is very sufficient for the construction and validation of prediction models. A larger sample size is valuable for developing a more robust prediction model, which has good generalization ability and good statistical efficacy for a wider population. However, the study was still subject to some limitations. First, the retrospective nature of the study may have introduced unavoidable selection bias, which limits the interpretation of the results. Second, the MIMIC data were obtained from a single center in the United States, which may affect the generalizability of the prediction model to other populations. The results may not be representative of the entire population of AP patients, although we attempted to provide detailed information in our study. Third, the study included AP patients in MIMIC-III and IV, which included hospitalized patients from 2001 to 2019. The population studied here is not consecutive and therefore different biases may have been introduced. As treatment regimens are developed and optimized, consistency of treatment regimens cannot be guaranteed, which may introduce some bias into the results. Fourth, radiological results in AP, specific chemoradiotherapy information, and medication dosage in vasopressors and mechanical ventilation may have an impact on our results, but the lack of radiological data in the database prevented us from performing further analyses. Fifth, the study lacked external validation. External validation is crucial to assess the generalizability and reliability of the model, especially when using data from a single center. Therefore, it would be important to perform further validation on an independent dataset in future studies to examine the robustness and generalization ability of the proposed model, which might greatly increase the impact of the current finding. Future research will need to explore other machine learning algorithms for predicting sepsis in AP patients.

## Conclusions

This study constructed and validated machine learning models to predict sepsis in patients with AP. The GBDT model, based on 13 predictive factors, showed promising performance in predicting sepsis in AP patients. A prediction model is a useful tool for the early identification of high-risk patients and timely clinical intervention.

### Electronic supplementary material

Below is the link to the electronic supplementary material.

**Supplementary Table 1** Variables with missing values below 20%

**Supplementary Table 2** comparations of the data before and after imputation

**Supplementary Figure 1**. The practical applicability of the GBDT model using SHAP

## Data Availability

The datasets generated and/or analyzed during the current study are available in the MIMIC III database (https://mimic.mit.edu/docs/iii/) and MIMIC IV database (https://mimic.mit.edu/docs/iv/).

## Abbreviations

AP: Acute pancreatitis
SIRS: systemic inflammatory response syndrome
SOFA: sequential organ failure assessment
qSOFA: quick-SOFA
SAPS II: simplified acute physiology score II
LR: logistic regression
AUC: area under the receiver
ROC: AUC of the operating characteristic curve
AKI: acute kidney injury
MIMIC III: Medical Information Mart for Intensive Care III
SBP: systolic blood pressure
DBP: diastolic blood pressure
GCS: Glasgow Coma Scale
CCI: charlson comorbidity index
INR: International Normalized Ratio
WBC: white blood cell
PT: prothrombin time
PTT: partial thromboplastin time
BUN: blood urea nitrogen
ICD: International Classification of Diseases
SVM: support vector machine
KNN: K-nearest neighbor
MLP: multi-layer perceptron
GBDT: gradient boosting decision tree
AdaBoost: adaptive enhancement algorithm
PPV: positive prediction value
NPV: negative prediction value
SD: standard deviations
