# Supplementary material for: Construction and validation of machine learning models for sepsis prediction in patients with acute pancreatitis
Source: BMC Surg. 2023 Sep 1;23:267. doi: 10.1186/s12893-023-02151-y (PMC10474758; doi:10.1186/s12893-023-02151-y)
Supplement: Supplementary file 1 — Supplementary Table 1 Variables with missing values below 20% [file 12893_2023_2151_MOESM1_ESM.docx]

**Supplementary Table 1 Variables with missing values below 20%**

| Variables | Missing rate, n (%) |
| --- | --- |
| PTT, sec | 285 (17.05%) |
| Bilirubin, mg/dL | 284 (16.99%) |
| INR | 261 (15.61%) |
| PT, sec | 261 (15.61%) |
| Marital status | 140 (8.37%) |
| Respiratory rate, breaths/minute | 30 (1.79%) |
| RDW, % | 30 (1.79%) |
| Temperature, °C | 29 (1.73%) |
| WBC, K/uL | 27 (1.61%) |
| Hemoglobin, g/dL | 27 (1.61%) |
| Calcium, mg/dL | 27 (1.61%) |
| Platelet count, K/uL | 25 (1.50%) |
| Hematocrit, % | 21 (1.26%) |
| BUN, mg/dL | 19 (1.14%) |
| Glucose, mg/dL | 19 (1.14%) |
| SBP, mmHg | 18 (1.08%) |
| Creatinine | 18 (1.08%) |
| Sodium, mEq/L | 18 (1.08%) |
| Chloride, mEq/L | 18 (1.08%) |
| Total bicarbonate, mEq/L | 18 (1.08%) |
| DBP, mmHg | 17 (1.02%) |
| GCS | 14 (0.84%) |
| Heart rate, bpm | 14 (0.84%) |
| SpO_2_, % | 14 (0.84%) |
| Age, years | 0 (0%) |
| Gender | 0 (0%) |
| Race | 0 (0%) |
| Insurance | 0 (0%) |
| Vasopressors | 0 (0%) |
| Mechanical ventilation | 0 (0%) |
| SOFA score | 0 (0%) |
| qSOFA | 0 (0%) |
| SAPS II | 0 (0%) |
| CCI | 0 (0%) |
| SIRS | 0 (0%) |
| Effusion | 0 (0%) |

Notes: PTT, partial thromboplastin time; INR, International Normalized Ratio; PT, prothrombin time; RDW, red blood cell distribution width; WBC, white blood cell; BUN, blood urea nitrogen; SBP, systolic blood pressure; DBP, diastolic blood pressure; GCS, Glasgow Coma Scale; SOFA, sequential organ failure assessment; qSOFA, quick-SOFA; SAPS, simplified acute physiology score; CCI, charlson comorbidity index; SIRS, systemic inflammatory response syndrome.
