# Supplementary material for: Construction and validation of machine learning models for sepsis prediction in patients with acute pancreatitis
Source: BMC Surg. 2023 Sep 1;23:267. doi: 10.1186/s12893-023-02151-y (PMC10474758; doi:10.1186/s12893-023-02151-y)
Supplement: Supplementary file 2 — Supplementary Table 2 comparations of the data before and after imputation [file 12893_2023_2151_MOESM2_ESM.docx]

**Supplementary Table 2 comparations of the data before and after imputation**

| Variables | Before imputation (n=1672) | After imputation (n=1672) | *P* |
| --- | --- | --- | --- |
| GCS, mean (SD) | 12.87 (3.32) | 12.88 (3.31) | 0.934 |
| Heart rate, bpm, mean (SD) | 99.35 (21.47) | 99.35 (21.38) | 0.997 |
| SBP, mmHg, mean (SD) | 127.51 (26.16) | 127.52 (26.03) | 0.997 |
| DBP, mmHg, mean (SD) | 70.75 (18.96) | 70.75 (18.88) | 0.995 |
| Respiratory rate, breaths/minute, mean (SD) | 21.14 (6.58) | 21.13 (6.53) | 0.964 |
| Temperature, mean (SD) | 36.87 (1.05) | 36.87 (1.04) | 0.989 |
| SpO_2_, %, mean (SD) | 96.20 (4.31) | 96.20 (4.29) | 1 |
| WBC, K/uL, mean (SD) | 14.20 (9.74) | 14.17 (9.67) | 0.933 |
| Platelet, K/uL, mean (SD) | 215.99 (130.78) | 215.75 (129.82) | 0.958 |
| Hemoglobin, g/dL, mean (SD) | 11.16 (2.29) | 11.17 (2.27) | 0.971 |
| RDW, %, mean (SD) | 15.13 (2.10) | 15.12 (2.08) | 0.94 |
| Hematocrit, %, mean (SD) | 33.43 (6.74) | 33.43 (6.70) | 0.993 |
| Bilirubin, mg/dL, mean (SD) | 2.76 (4.69) | 2.67 (4.30) | 0.565 |
| Creatinine, mg/dL, M [Q_1_, Q_3_] | 1.10 (0.70, 1.80) | 1.10 (0.70, 1.80) | 0.858 |
| INR, mean (SD) | 1.63 (1.15) | 1.62 (1.06) | 0.749 |
| PT, sec, mean (SD) | 17.09 (9.71) | 17.02 (8.94) | 0.816 |
| PTT, sec, mean (SD) | 36.09 (18.96) | 35.62 (17.33) | 0.475 |
| BUN, mg/dL, mean (SD) | 29.36 (25.45) | 29.38 (25.33) | 0.981 |
| Glucose, mg/dL, mean (SD) | 154.62 (112.61) | 154.49 (111.97) | 0.974 |
| Calcium, mg/dL, mean (SD) | 7.02 (2.49) | 7.03 (2.48) | 0.909 |
| Sodium, mEq/L, mean (SD) | 138.37 (5.65) | 138.37 (5.62) | 0.999 |
| Chloride, mEq/L, mean (SD) | 105.02 (7.19) | 105.02 (7.15) | 0.989 |
| Bicarbonate, mEq/L, mean (SD) | 21.26 (5.59) | 21.26 (5.56) | 0.969 |

Notes: PTT, partial thromboplastin time; INR, International Normalized Ratio; PT, prothrombin time; RDW, red blood cell distribution width; WBC, white blood cell; BUN, blood urea nitrogen; SBP, systolic blood pressure; DBP, diastolic blood pressure; GCS, Glasgow Coma Scale.
