# Supplementary figures and images for: Construction and validation of machine learning models for sepsis prediction in patients with acute pancreatitis
Source: BMC Surg. 2023 Sep 1;23:267. doi: 10.1186/s12893-023-02151-y (PMC10474758; doi:10.1186/s12893-023-02151-y)

Supplementary Figure 1. The practical applicability of the GBDT model using SHAP

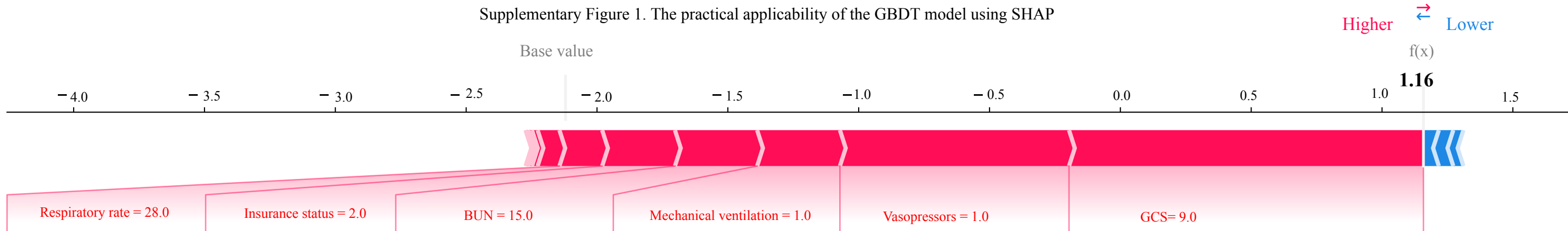

Supplement: Supplementary file 3 — Supplementary Figure 1. The practical applicability of the GBDT model using SHAP [file 12893_2023_2151_MOESM3_ESM.pdf]
